# Recombinant CTRP9 administration attenuates neuroinflammation via activating adiponectin receptor 1 after intracerebral hemorrhage in mice

**DOI:** 10.1186/s12974-018-1256-8

**Published:** 2018-07-30

**Authors:** Lianhua Zhao, Shengpan Chen, Prativa Sherchan, Yan Ding, Wei Zhao, Zaiyu Guo, Jing Yu, Jiping Tang, John H. Zhang

**Affiliations:** 1 Department of Neurology, Tianjin TEDA Hospital, Tianjin, China; 2 Department of Physiology and Pharmacology, Loma Linda University, 11041 Campus St, Loma Linda, CA 92354 USA; 3 Department of Neurosurgery, Affiliated Haikou Hospital, Xiangya School of Medicine, Central South University, Haikou, China

**Keywords:** C1q/TNF-related protein 9, Adiponectin receptor 1, Intracerebral hemorrhage, Neuroinflammation, Adenosine monophosphate-activated protein kinase

## Abstract

**Background:**

Neuroinflammation is a crucial factor contributing to neurological injuries after intracerebral hemorrhage (ICH). C1q/TNF-related protein 9 (CTRP9), an agonist of adiponectin receptor 1 (AdipoR1), has recently been shown to reduce inflammatory responses in systemic diseases. The objective of this study was to investigate the protective role of CTRP9 against neuroinflammation after ICH in a mouse model and to explore the contribution of adenosine monophosphate-activated protein kinase (AMPK)/nuclear factor kappa B (NFκB) pathway in AdipoR1-mediated protection.

**Methods:**

Adult male CD1 mice (*n* = 218) were randomly assigned to different groups for the study. ICH was induced via intrastriatal injection of bacterial collagenase. Recombinant CTRP9 (rCTRP9) was administered intranasally at 1 h after ICH. To elucidate the underlying mechanism, AdipoR1 small interfering ribonucleic acid (siRNA) and selective phosphorylated AMPK inhibitor Dorsomorphin were administered prior to rCTRP9 treatment. Brain edema, short- and long-term neurobehavior evaluation, blood glucose level, western blot, and immunofluorescence staining were performed.

**Results:**

Endogenous CTRP9 and AdipoR1 expression was increased and peaked at 24 h after ICH. AdipoR1 was expressed by microglia, neurons, and astrocytes. Administration of rCTRP9 reduced brain edema, improved short- and long-term neurological function, enhanced the expression of AdipoR1 and p-AMPK, and decreased the expression of phosphorylated NFκB and inflammatory cytokines after ICH. The protective effects of rCTRP9 were abolished by administration of AdipoR1 siRNA and Dorsomorphin.

**Conclusions:**

Our findings demonstrated that administration of rCTRP9 attenuated neuroinflammation through AdipoR1/AMPK/NFκB signaling pathway after ICH in mice, thereby reducing brain edema and improving neurological function after experimental ICH in mice. Therefore, CTRP9 may provide a potential therapeutic strategy to alleviate neuroinflammation in ICH patients.

**Electronic supplementary material:**

The online version of this article (10.1186/s12974-018-1256-8) contains supplementary material, which is available to authorized users.

## Background

Spontaneous intracerebral hemorrhage (ICH) is a severe type of stroke with high morbidity and mortality, accounting for about 15% of all stroke patients [1]. Intracerebral hematoma oppresses surrounding brain tissue is considered primary brain injury. Then, red blood cell debris and blood components breakdown can initiate the secondary brain injury and persist for several days to weeks in the perihematoma area [2]. Increasing evidence indicates that inflammatory response that occurs in the early stage after ICH is a key factor leading to secondary brain injury induced by ICH. Treatment to inhibit neuroinflammation after the onset of ICH can provide critical therapeutic strategy to improve outcomes after ICH.

Adiponectin is a major adipocyte-derived hormone that exerts its beneficial physiological effects such as regulation of metabolism, anti-atherosclerosis, and anti-inflammatory effects by activating its receptors [3–5]. Adiponectin receptor 1 (AdipoR1) is the main isoform of adiponectin receptors. Activation of AdipoR1 has been shown to have anti-inflammatory effects in the brain [6]. The C1q/TNF-related protein (CTRP) family is a novel analog of adiponectin, among which the globular C1q domain of CTRP9 has the highest degree of amino acid sequence homology with adiponectin. Studies have shown that CTRP9 has a protective effect against inflammation after cardiac ischemia [7, 8]. However, its role in stroke has not been investigated.

Adenosine monophosphate-activated protein kinase (AMPK) signaling system plays a key role in cellular and organismal survival by its ability to maintain metabolic homeostasis. Activation of AMPK has been shown to inhibit inflammatory response in various injury models while suppression of AMPK activity was associated with increased inflammation [9, 10]. Furthermore, the nuclear factor kappa B (NFκB) signaling system is one of the primary pathways involved in pro-inflammatory responses. Previous studies have shown that activation of AMPK signaling can downregulate the function of NFκB system [11–13]. To date, the anti-inflammatory role of CTRP9 in the brain has not been studied.

In the present study, we hypothesized the following three points: (1) rCTRP9 administration by intranasal route improves neurological deficits and brain edema after ICH; (2) rCTRP9 administration attenuates the production of inflammatory mediators following ICH; (3) the anti-inflammatory effect of rCTRP9 is mediated through the AdipoR1/AMPK/NFκB pathway after ICH.

## Methods

### Animals

Eight-week-old male CD1 mice (weight 30–40 g, Charles River, Wilmington, MA) were housed in a 12-h light/dark cycle at a controlled temperature and humidity with unlimited access to food and water. All experimental procedures were performed following the study protocol approved by the Institutional Animal Care and Use Committee (IACUC) at Loma Linda University in accordance with the National Institutes of Health Guide for the Care and Use of Laboratory Animals.

### Experimental design

In the present study, all mice were randomly assigned to the following experiments. The following five separate experiments are shown in the timeline of experimental design (Additional file 1: Figure S1). The summary of experimental groups, animal numbers, and mortality rate in the study are listed in a table (Additional file 2: Table S1).

#### Experiment 1

To evaluate time course expression of endogenous CTRP9 and AdipoR1 after ICH, 36 mice were randomly divided into 6 groups for western blot: sham, ICH after 3, 6, 12, 24, and 72 h (*n* = 6/group). Additional two mice were used for immunofluorescence staining at 24 h post-ICH. Western blot was used to detect the expression of CTRP9 and AdipoR1 in the ipsilateral (right) hemisphere. Immunofluorescence staining was performed to localize AdipoR1 on neurons, astrocytes, and microglia.

#### Experiment 2

To determine the best treatment dosage for rCTRP9, 30 mice were randomly divided into 5 groups, *n* = 6/group: sham, ICH + vehicle (PBS), ICH + rCTRP9 (0.03 μg/g), ICH + rCTRP9 (0.1 μg/g), ICH+ rCTRP9 (0.3 μg/g). rCTRP9 was administered intranasally at 1 h post-ICH. Neurobehavioral tests and brain water content (BWC) were assessed at 24 h post-ICH. Blood glucose level was measured starting 1, 2, 3, 6, and 24 h after ICH.

#### Experiment 3

To determine the presence of rCTRP9 in the brain after intranasal administration, mice (*n* = 24) were divided into 4 groups, *n* = 6/group: naive, naïve+rCTRP9 (0.1 μg/g), ICH, ICH + rCTRP9 (0.1 μg/g). Based on results from experiment 2, medium dosage (0.1 μg/g) of rCTRP9 was chosen for the study. Neurobehavioral tests and western blot to measure rCTRP9 expression in ipsilateral (right) hemisphere were performed at 24 h post-ICH. Next, for the effects of the drug at 72 h after ICH, 18 mice were divided into 3 groups, *n* = 6/group: sham, ICH + vehicle (PBS), ICH + rCTRP9 (0.1 μg/g). Neurobehavioral tests and BWC were evaluated at 72 h post-ICH.

#### Experiment 4

To assess long-term neurobehavioral function, 24 mice were divided into 3 groups: sham, ICH + vehicle (PBS), ICH + rCTRP9 (0.1 μg/g), *n* = 8/group. The foot fault test and rotarod test were performed on days 7, 14, and 21 post-ICH. Morris water maze test was conducted on days 21–25 post-ICH.

#### Experiment 5

To verify the efficacy of AdiporR1 siRNA to knockdown the expression of AdipoR1, 24 mice were divided into 4 groups, *n* = 6/group: naive, naïve+AdipoR1 small interfering ribonucleic acid (siRNA), ICH, ICH + AdipoR1 siRNA. Next, to verify the anti-inflammatory mechanism of rCTRP9, mice (*n* = 42) were divided into seven groups: sham, ICH + vehicle (PBS), ICH + rCTRP9 (0.1 μg/g), ICH + rCTRP9 + AdipoR1 siRNA, ICH + rCTRP9 + scramble siRNA (Scr siRNA), ICH + rCTRP9 + Dorsomorphin, ICH + rCTRP9 + DMSO (5 μl of 5% dimethyl sulfoxide in PBS), *n* = 6/group. Neurobehavioral tests and western blot were performed at 24 h post-ICH.

### ICH model

ICH was induced by intrastriatal injection of bacterial collagenase into the right basal ganglia as previously described [14]. Briefly, mice were anesthetized with ketamine (100 mg/kg) and xylazine (10 mg/kg) (2:1 vol/vol, intraperitoneal injection) and positioned prone in a stereotaxic head frame. Bacterial collagenase (0.075 units, type VII-S; Sigma-Aldrich, St. Louis, MO) was dissolved in 0.5 μL PBS and infused into the right basal ganglia at a rate of 0.1667 μL/min, using an infusion pump (Stoelting, IL). The needle was left for an additional 5 min to prevent possible leakage of the collagenase solution, and withdrawn slowly at a rate of 1 mm/min. After removal of the needle, the cranial burr hole was sealed with bone wax, the incision was sutured, and 0.4 mL of normal saline was injected subcutaneously, and the mice were allowed to recover. The sham operation was performed with PBS only. All ICH animals with hematoma and neurological deficits were included. Animals that died before experimental endpoints reached were replaced with new animals. Animals with no hematoma were excluded.

### Drug administration

Recombinant CTRP9 (rCTRP9) (Novus, CO) dissolved in PBS was administered intranasally at 1 h post-ICH as previously reported [15]. Briefly, rCTRP9 was diluted in PBS, and three different doses of rCTRP9 (0.03, 0.1, and 0.3 μg/g) were treated. Nasal administration of rCTRP9 was performed at 1 h after ICH induction. A total volume 20 μL of rCTRP9 was administered intranasally. Mice, still under anesthesia, were placed in a supine position, and rCTRP9 was administered alternating drops (5 μL/drop) every 2 min between the left and right nares over a period of 20 min. The ICH + vehicle group received an equal volume of phosphate-buffered saline (PBS).

### Intracerebroventricular injection

AdipoR1 siRNA and scramble siRNA (scr siRNA) (Life Technologies, NY) were delivered via intracerebroventricular (ICV) injection 48 h before ICH as previously described [16]. Briefly, AdipoR1 SiRNA or scr siRNA (100 pmol/mouse) was dissolved in sterile RNAs free resuspension buffer according to the manufacturer’s instructions (Life Technologies, NY). The ICV injections into the left ventricle were performed using the following coordinates relative to bregma (0.22 mm posterior, 1.0 mm lateral, and 2.25 mm deep) at a rate of 0.667 μL/min at 48 h prior to surgery. After waiting for 5 min, the needle was removed over a period of 3 min. The burr hole was sealed with bone wax. Dorsomorphin (5 μg/mouse, Sigma, MO) was dissolved in DMSO and given by ICV injection into left ventricle as described above at 30 min before ICH surgery.

### Short-term neurobehavior assessment

Short-term neurobehavioral tests were performed with modified Garcia score test, forelimb placement test, and corner turn test by an experienced investigator who was blinded to the experimental groups at 24 and 72 h post-ICH, as previously reported [17]. Garcia score assessment with a 21-point score evaluating spontaneous activity, axial sensation, vibrissae proprioception, symmetry of limb movement, lateral turning, forelimb walking, climbing, and grabbing was conducted. The forelimb placement test was used to investigate the animals’ responsiveness to vibrissae stimulation; the placement of the left and right forelimbs was investigated for 10 consecutive trials. Then, left forelimb placement was calculated as left forelimb placement/(left forelimb placement + right forelimb placement) × 100%. For the corner turn test, animals were allowed to advance into a 30° corner and exit by turning either to the left or right. Choice of turning was recorded for a total of 10 trials, and a score was calculated as number of left turns/all trials × 100.

### Brain water content measurement

The brain water content (BWC) was measured as previously reported using the wet/dry method [17]. Mice were euthanized under deep anesthesia and the brains were removed immediately and divided into five parts: ipsilateral and contralateral cortex, ipsilateral and contralateral basal ganglia, and cerebellum. All brain samples were weighed on analytical microbalance (APX-60 Denver Instrument, NY) to obtain the wet weight (WW). Then the samples were dried at 100 °C for 48 h to obtain the dry weight (DW). Brain water content (%) was calculated using the following formula: (WW − DW)/WW*100%.

### Blood glucose measurement

Plasma glucose level was measured by blood glucose meter (Ascensia Diabetes Care US Inc., NJ) in the blood collected from tail vein before ICH and at 1, 2, 3, 6, and 24 h after ICH in each group.

### Long-term neurobehavior assessment

Foot-fault and rotarod test were performed at the first, second, and third weeks post-ICH to assess sensorimotor function, coordination, and balance as previously described [18]. Water maze tests, including swim distance and escape latency, were performed at days 21 to 25 post-ICH to evaluate memory and spatial learning as previously described [19]. Morris water maze test probe quadrant duration was evaluated on day 25 post-ICH.

### Western blot analysis

Western blotting was performed as described previously [20]. Briefly, the brain protein samples were prepared using Ripa Lysis buffer (Bio-Rad, CA), and equal amounts of protein were run on an SDS-PAGE gel. After being electrophoresed and transferred to a nitrocellulose membrane, the membrane was blocked and incubated with primary antibody overnight at 4 °C. The following primary antibodies were used: AdipoR1 (1:1000, ab126611), p-AMPKα (1:1000, ab133448), AMPKα (1:1000, ab32047), p-NFκB (1:1000, ab86299), NFκB (1:2000, ab16502), tumor necrosis factorα (TNFα) (1:1000, ab6671), interleukin-6 (IL-6) (1:1000, ab6672) (all from Abcam, MA), CTRP9 (1:500, NBP2–46834, Novus, CO), and adaptor protein, phosphotyrosine interacting with PH domain and leucine zipper 1 (APPL1) (1:1000, sc-271,901, Santa Cruz Biotechnology, CA). β-actin was used as an internal loading control. The respective secondary antibodies were incubated for 1 h at room temperature. The bands were probed with an ECL Plus chemiluminescence reagent Kit (Amersham Biosciences, Arlington Heights, PA) and visualized with the image system (Bio-Rad, Versa Doc, model 4000). Relative density of the protein immunoblot images were analyzed by ImageJ software (ImageJ 1.4, NIH, USA).

### Immunofluorescence staining

At 24 h following ICH, mice were perfused under deep anesthesia with cold PBS (pH 7.4). Mice were then infused with 10% formalin. Brains were then removed and fixed in formalin at 4 °C for a minimum of 3 days. Samples were then dehydrated with 30% sucrose in PBS (pH 7.4). The frozen coronal slices (8 μm thick) were sectioned in cryostat (CM3050S; Leica Microsystems). Double immunofluorescence staining was performed as previously described [20]. The primary antibodies included anti-AdipoR1 (1:200, ab126611), anti-Iba-1 (1:200, ab178847), anti-NeuN (1:200, ab177487), and anti-GFAP (1:200, ab16997) (all from Abcam, MA).

### Statistical analysis

All animals were randomly assigned to different groups. All the experimental tests were blinded to the surgeons and researchers who did the experiments and analyzed the research data. All tests for exploratory studies were performed two-sided. All data were expressed as the mean and standard deviation (mean ± SD). Statistical analysis was performed with GraphPad Prism (Graph Pad Software, San Diego, CA). One-way analysis of variance (ANOVA) is followed by multiple comparisons between groups using Tukey’s post hoc test. Two-way repeated measures ANOVA was used to analyze the long-term neurobehavioral functions over time. Statistical significance was set at *p* < 0.05.

## Results

### Animal mortality rate

A total of 218 mice were used of which 56 were sham and 162 mice underwent ICH induction. None of the sham mice died, and the mortality rate in ICH group was 9.2% (15/162). Three mice were excluded from the study because of no hemorrhage. An additional file shows this in more details (Additional file 2: Table S1).

### Endogenous CTRP9 and AdipoR1 expression were increased after ICH

**Fig. 1:**
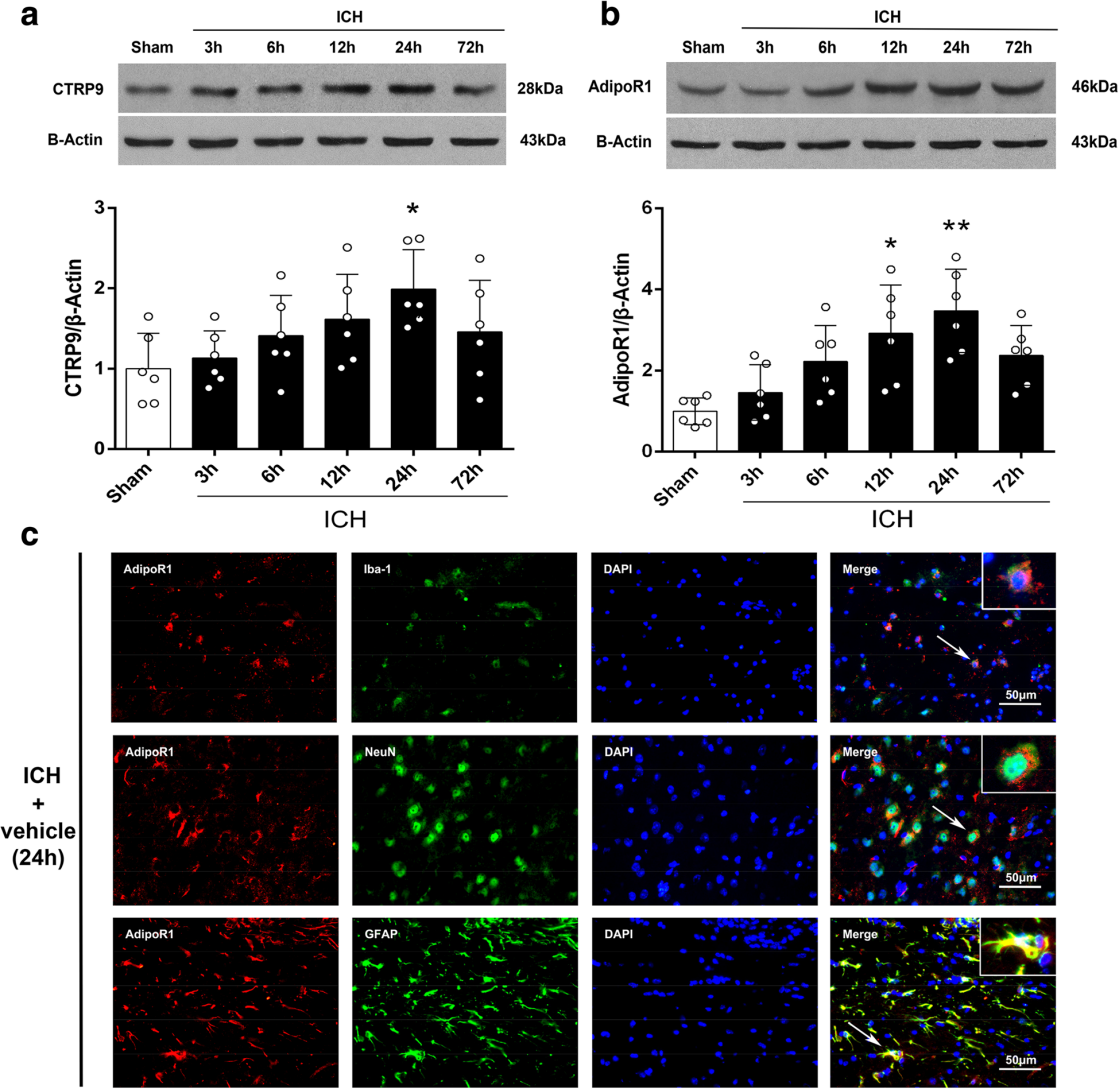
Expression of C1q/TNF-related protein 9 (CTRP9) and adiponectin receptor 1 (AdipoR1) after intracerebral hemorrhage (ICH). **a** Representative western blot images and quantitative analyses of CTRP9 time course after ICH. **b** Representative western blot images and quantitative analyses of AdipoR1 time course after ICH. Values are expressed as mean ± SD. *p < 0.05, **p < 0.01 vs. sham group. *N* = 6. **c** Double immunofluorescence staining for AdipoR1 (red) in microglia (Iba-1, green), neurons (NeuN, green), and astrocytes (GFAP, green) in right basal cortex 24 h after ICH. Scale bar = 50 μm. *N* = 2. DAPI, 4′,6-diamidino-2-phenylindole; Iba-1, ionized calcium binding adaptor molecule-1; NeuN, neuronal nuclear; GFAP, glial fibrillary acidic protein

The expression of endogenous CTRP9 and AdipoR1 was increased after ICH with a peak at 24 h compared to sham group and then decreased at 72 h after ICH (*p* < 0.05, Fig. 1a, b). Immunofluorescence staining showed that AdipoR1 was expressed in microglia (Iba-1), neurons (NeuN), and astrocytes (GFAP) at 24 h post-ICH (Fig. 1c).

### rCTRP9 attenuated neurobehavioral deficits and reduced brain edema at 24 h after ICH

**Fig. 2:**
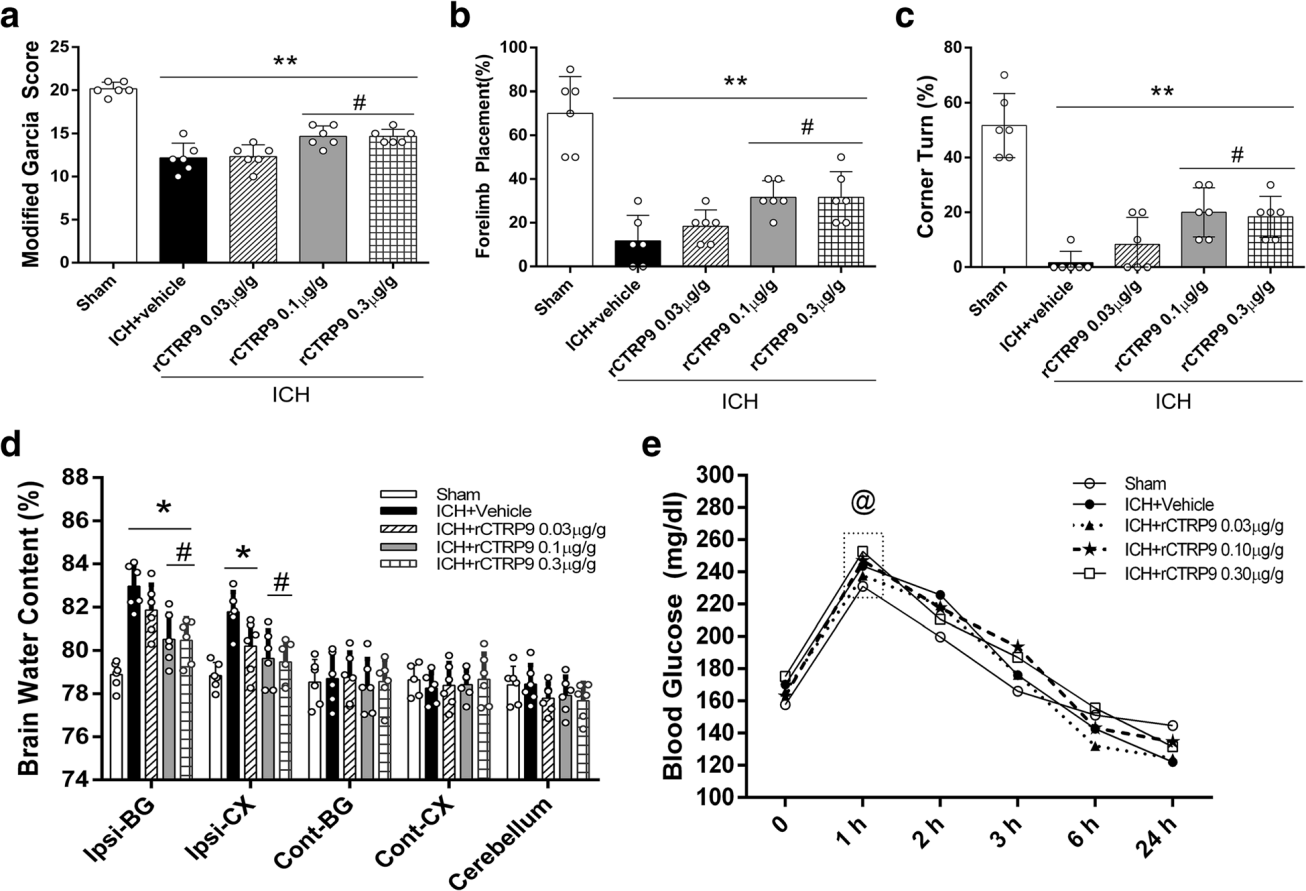
The effects of different doses of rCTRP9 on neurobehavior tests, brain water content (BWC), and blood glucose at 24 h post-ICH. **a** Modified Garcia test, **b** forelimb placement test, **c** corner turn test, and **d** BWC at 24 h post-ICH. **e** Blood glucose levels measured at 0, 1, 2, 3, 6, and 24 h post-ICH. Values are expressed as mean ± SD. *p < 0.05, **p < 0.01 vs. sham group; #*p* < 0.05 vs. ICH + vehicle group; @*p* < 0.05 vs. before ICH (0 h); *N* = 6

The neurobehavioral score was significantly decreased in vehicle group compared with the sham group at 24 h post-ICH. Administration of medium dose and high dose of rCTRP9 significantly improved neurological function and decreased BWC in the right basal ganglia and cortex compared with ICH + vehicle group (*p* < 0.05, Fig. 2a–d).

### rCTRP9 did not change blood glucose level after ICH

Blood glucose level increased significantly at 1 h post-surgery in all groups compared to baseline before surgery (*p* < 0.05), then continued to decline and reached nadir at 24 h post-ICH (Fig. 2e). There was no significant difference in blood glucose levels between experimental groups when compared at the same time point (*p* > 0.05). Based on above results, medium dosage rCTRP9 was used in the following experiments.

### Nasal rCTRP9 administration increased the brain expression level of CTRP9 in naïve and ICH mice

**Fig. 3:**
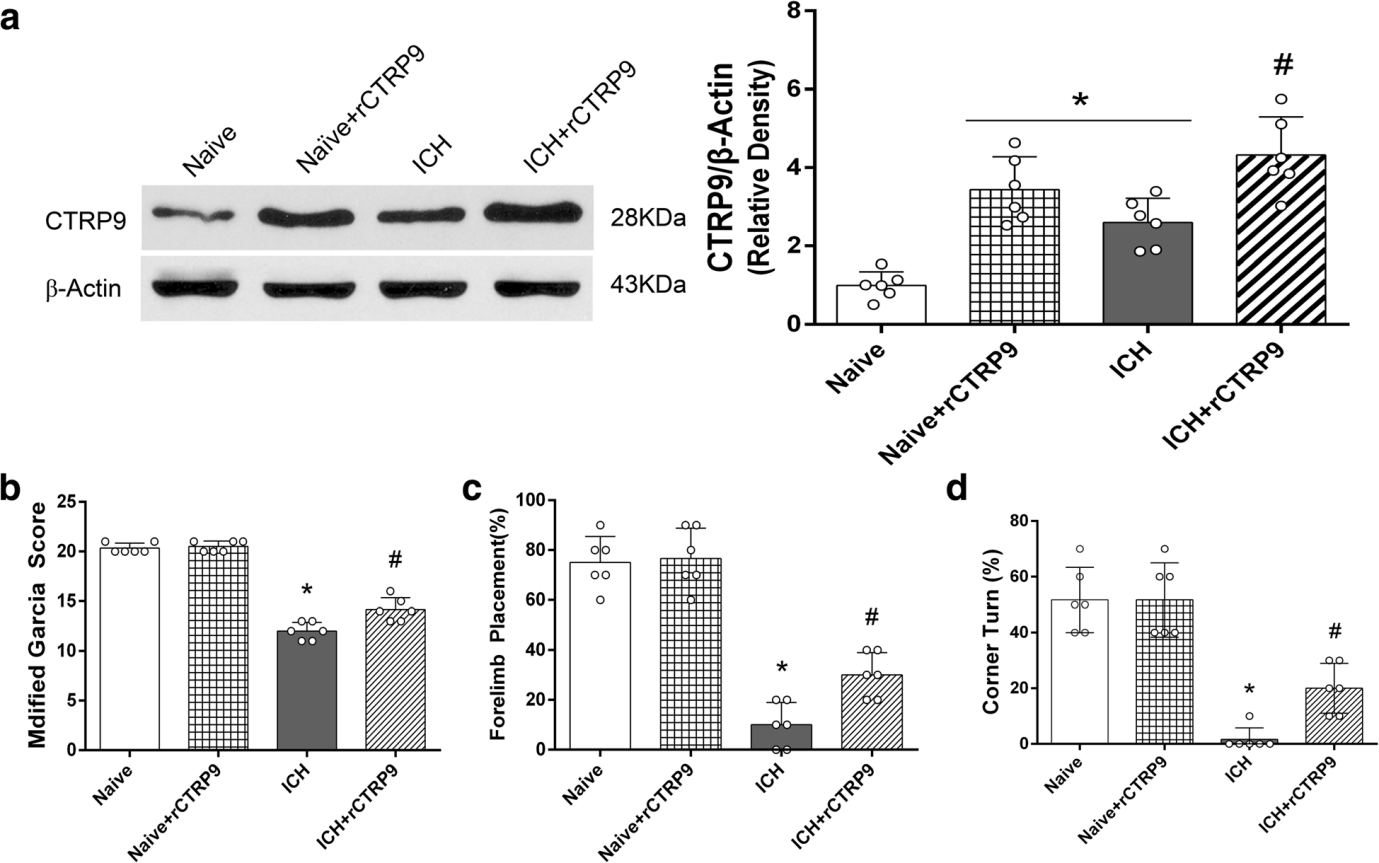
Brain expression of CTRP9 and neurological function evaluation after nasal administration of rCTRP9 at 24 h post-ICH. **a** Representative western blot images and quantitative analyses of CTRP9. Nasal administration of rCTRP9 increased the expression level of CTRP9 in naïve mice and ICH mice at 24 h post-ICH. **b** Modified Garcia test, **c** forelimb placement test, and **d** corner turn test showed that nasal administration of rCTRP9 improved neurological function at 24 h post-ICH. Values are expressed as mean ± SD. *p < 0.05 vs. naive group; #*p* < 0.05 vs. ICH group; *N* = 6

At 24 h after nasal administration of rCTRP9, the expression of CTRP9 in naïve+CTRP9 group was significantly increased compared with naïve group. The expression level of CTRP9 was significantly increased in ICH + rCTRP9 group compared to ICH group at 24 h after ICH (*p* < 0.05, Fig. 3a). The neurobehavioral score in the ICH + rCTRP9 group was significantly improved compared with ICH group (*p* < 0.05, Fig. 3b–d).

### rCTRP9 attenuated neurobehavioral deficits and reduced brain edema at 72 h after ICH

**Fig. 4:**
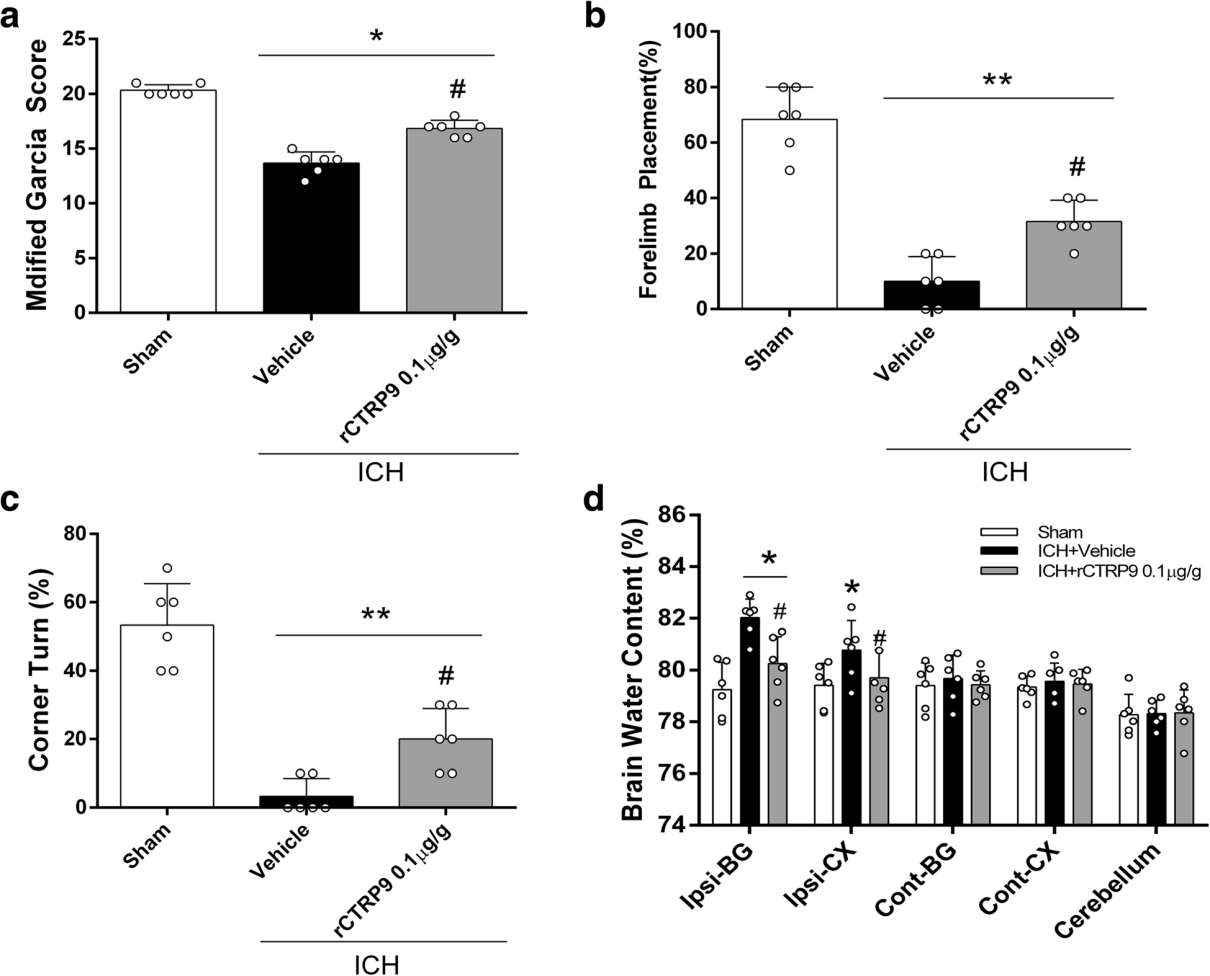
rCTRP9 improved neurological deficits and decreased brain water content (BWC) at 72 h post-ICH. **a** Modified Garcia test, **b** forelimb placement test, **c** corner turn test, and **d** BWC at 72 h post-ICH. Values are expressed as mean ± SD. *p < 0.05, **p < 0.01 vs. sham group; #*p* < 0.05 vs. ICH + vehicle group; *N* = 6

The neurobehavioral score was significantly improved, and brain edema in the right basal ganglia and cortex was reduced with rCTRP9 treatment compared with ICH + vehicle group at 72 h after ICH (*p* < 0.05, Fig. 4a–d).

### rCTRP9 improved long-term neurobehavioral outcomes after ICH

**Fig. 5:**
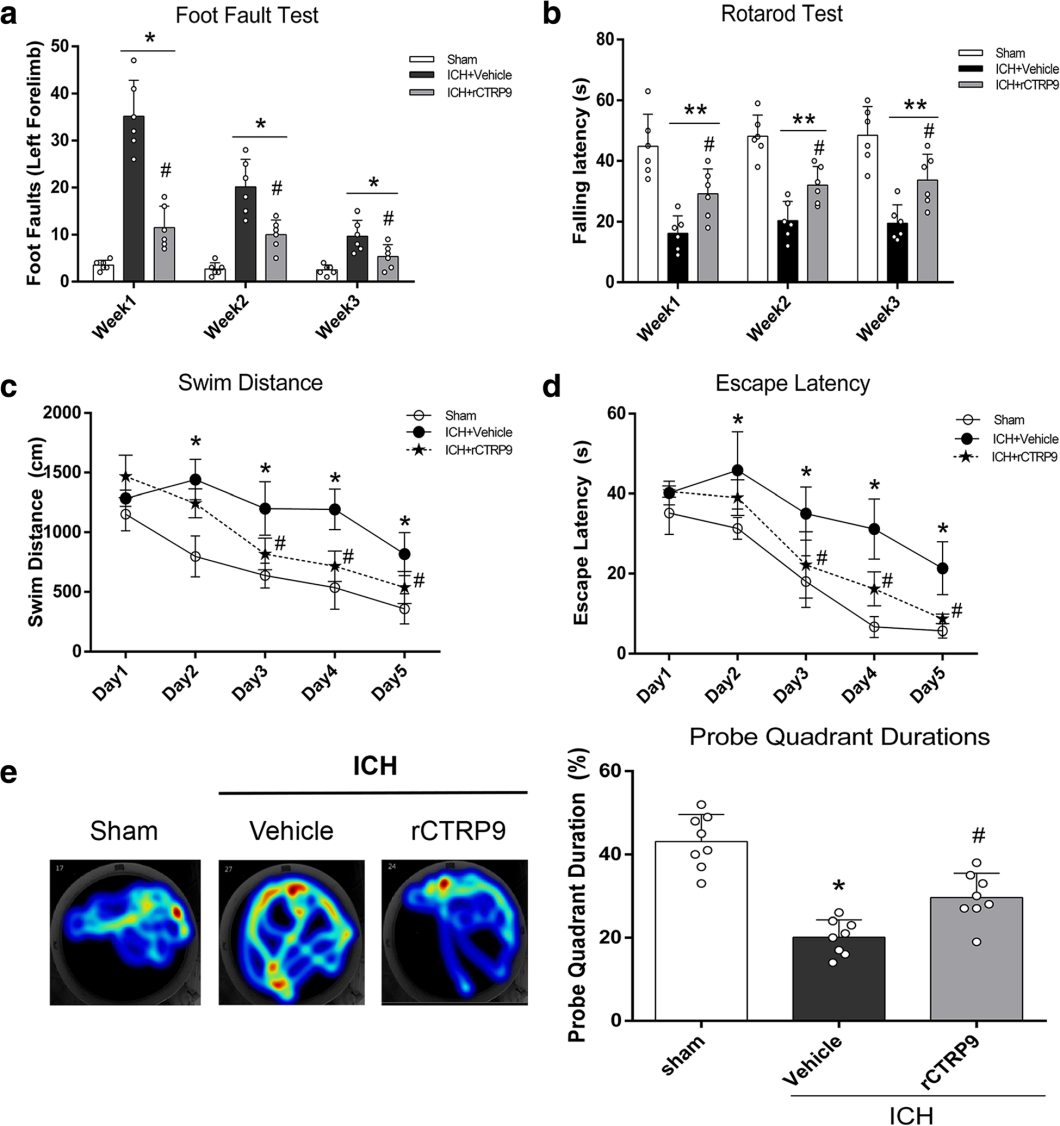
rCTRP9 improved long-term neurobehavioral function after ICH. **a** Foot fault test and rotarod test on days 7, 14, and 21 after ICH. **b** Morris water maze indicated by swim distance and escape latency on 21–25 days after ICH. **c** Morris water maze test probe quadrant duration on day 25 post-ICH. Values are expressed as mean ± SD. *p < 0.05, **p < 0.01 vs. sham group; #*p* < 0.05 vs. ICH + vehicle group. *N* = 8

In the foot fault test, ICH + vehicle group showed significantly increased foot faults compared to sham group and in the rotarod test, ICH + vehicle group had shorter latency to fall compared to sham group at 1, 2, and 3 weeks after ICH. Animals with rCTRP9 treatment showed significant improvement in both tests (*p* < 0.05; Fig. 5a, b).

In the water maze test, ICH + vehicle group showed significantly longer swim distance and escape latency. The rCTRP9 treatment group had significantly improved performance on days 3 to 5 of testing (*p* < 0.05; Fig. 5c, d). In the probe trial, the ICH + vehicle group spent less time in the target quadrant compared with sham group, while rCTRP9 treatment markedly increased the time spent in the probe quadrant (*p* < 0.05, Fig. 5e).

### AdipoR1 siRNA aggravated neurological deficits and decreased AdipoR1 expression in naïve and ICH mice

**Fig. 6:**
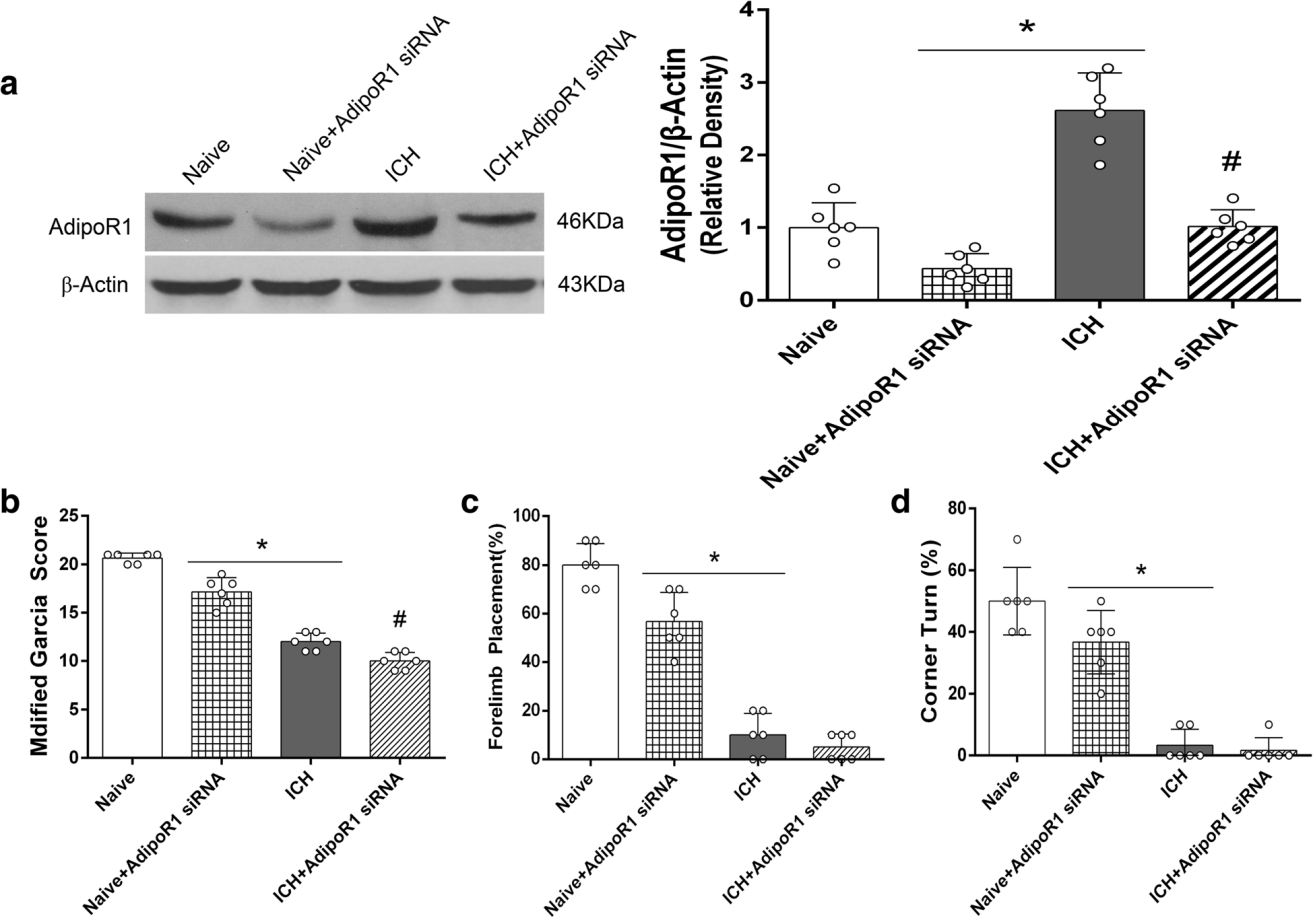
AdipoR1 expression and neurological function evaluation after AdipoR1 siRNA administration in naïve and ICH mice. **a** Representative western blot images and quantitative analyses of AdipoR1 expression. AdipoR1 siRNA decreased the expression of AdipoR1 in naïve mice and ICH mice at 24 h post-ICH. **b** Modified Garcia test, **b** forelimb placement test, and **c** corner turn test showed that AdipoR1 siRNA aggravated neurological deficits in naïve mice and ICH mice at 24 h post-ICH. Values are expressed as mean ± SD. *p < 0.05 vs. naive group; #*p* < 0.05 vs. ICH group; *N* = 6

AdipoR1 siRNA administration significantly decreased the expression of AdipoR1 and reduced neurobehavioral score in naïve+AdipoR1 siRNA group compared with naïve group. Similarly, the expression level of AdipoR1 was significantly decreased, and neurobehavior score was reduced in ICH + AdipoR1 siRNA group compared with ICH group (*p <* 0.05, Fig. 6a–d).

### AdipoR1 siRNA and Dorsomorphin abolished the anti-inflammatory effects of rCTRP9

**Fig. 7:**
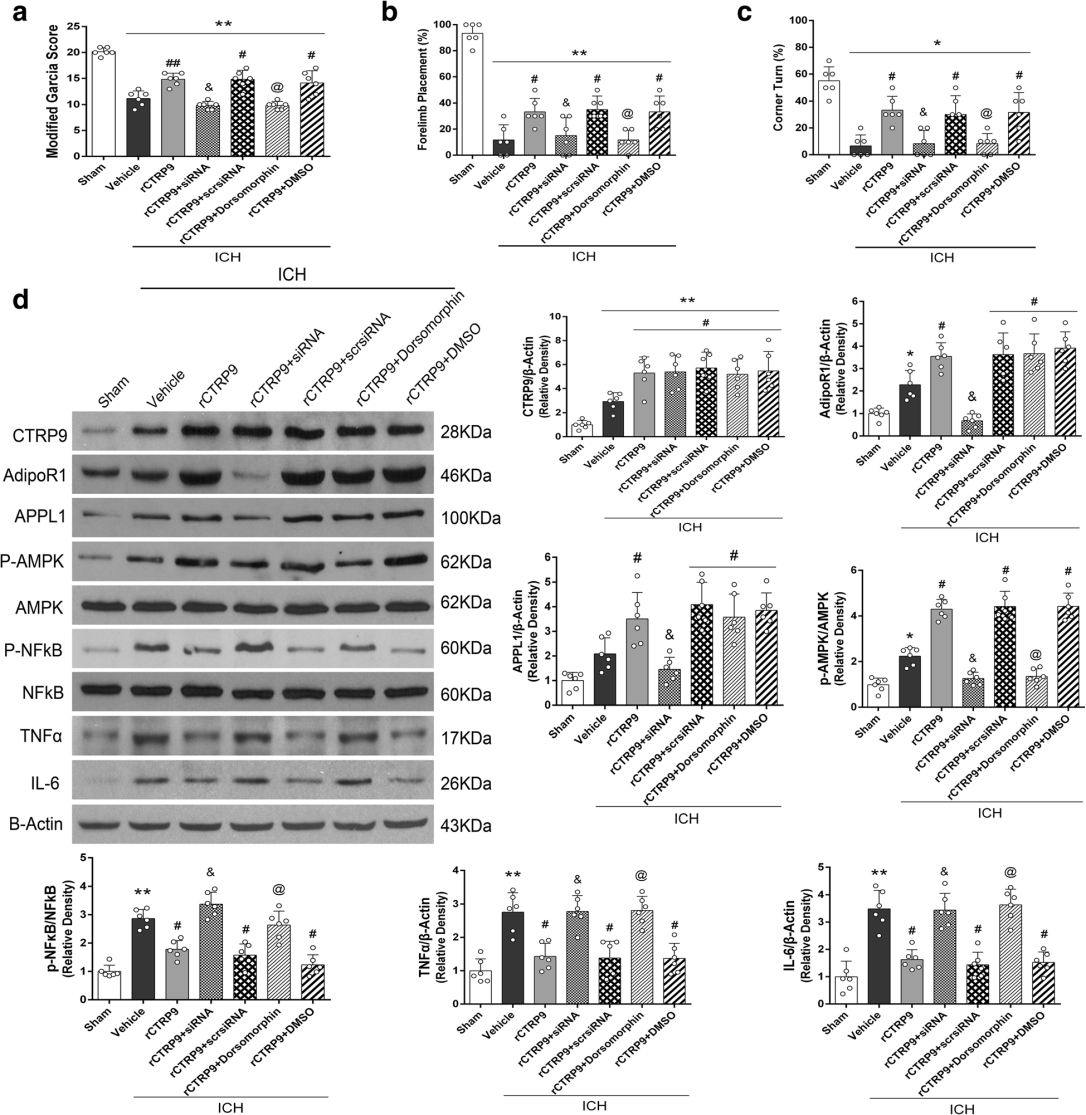
Knockdown of AdipoR1 and p-AMPK abolished improvement in neurological function and anti-inflammatory effects of rCTRP9 at 24 h post-ICH. **a** Modified Garcia test, **b** forelimb placement test, **c** corner turn test, and **d** representative western blot images and quantitative analyses of CTRP9, AdipoR1, APPL1, p-AMPK/AMPK, p-NFκB/NFκB, TNF-α, and IL-6 at 24 h post-ICH. Values are expressed as mean ± SD. *p < 0.05, **p < 0.01 vs. sham group; #*p* < 0.05, ##*p* < 0.01 vs. ICH + vehicle group; &*p* < 0.05 vs. rCTRP9 + Scr siRNA, and @*p* < 0.05 vs. rCTRP9 + DMSO group. Scr, scramble; siRNA, small interfering RNA. *N* = 6

AdipoR1 siRNA but not scramble siRNA reversed neurobehavioral improvement observed in ICH + rCTRP9 mice at 24 h post-ICH. Likewise, Dorsomorphin significantly decreased neurobehavioral score compared with ICH + rCTRP9 + DMSO group (*p* < 0.05, Fig. 7a–c).

The expression of CTRP9 was significantly increased in all ICH groups that received rCTPR9 via intranasal administration compared to sham and ICH + vehicle groups. The expression of AdipoR1, APPL1, and p-AMPK increased after rCTRP9 administration in ICH + rCTRP9 group compared to ICH + vehicle group. AdipoR1 siRNA significantly decreased the expression of AdipoR1, APPL1, and p-AMPK, while increasing the expression of p-NFκB, TNFα, and IL-6 when compared with ICH + rCTRP9 + Scr siRNA group. In addition, Dorsomorphin intervention significantly suppressed p-AMPK expression, while increasing p-NFκB, TNFα, and IL-6 expression when compared with ICH + rCTRP9 + DMSO group (*p* < 0.05; Fig. 7d).

## Discussion

In the present study, we first showed that the expression of CTRP9 and AdipoR1 increased in the brain at 24 h after ICH. Moreover, we found that rCTRP9 administration improved both short- and long-term neurobehavioral outcomes, alleviated brain edema, and attenuated neuroinflammation after ICH, which were accompanied by an increase in AdipoR1 and p-AMPK expression and a decrease in pro-inflammatory factors TNFα and IL-6 expression. Additionally, we determined that AdipoR1/AMPK/NFκB signaling pathway was involved in the anti-inflammatory effects of rCTRP9 after ICH. These findings suggest that rCTRP9 administration possibly improved outcomes after ICH at least in part by attenuating neuroinflammation through AdipoR1/AMPK/NFκB signaling pathway. The schematic mechanism was shown in a figure (Additional file 3: Figure S2).

Adiponectin is a biologically active polypeptide secreted by adipocytes and plays important roles in the regulation of energy homeostasis and insulin sensitivity. However, adiponectin knockout mice showed a relatively modest phenotype in the absence of diet or metabolic stress [21]. These findings suggested that there was a potential effective compensatory mechanism involved. CTRP9 shares the highest degree of sequence identity with adiponectin at the presumed functional globular domain, and has similar biochemical properties to adiponectin [22]. Existing research shows that CTRP9 has a variety of beneficial functions such as relaxing blood vessels, protecting the vascular endothelium, regulating metabolism, and reducing the expression of inflammatory factors [23, 24]. In addition, CTRP9 has also been shown to decrease myocardial infarct size by inhibiting the inflammatory response [25]. However, its role in the brain has never been studied.

In the present study, we demonstrated for the first time that CTRP9 has anti-inflammatory effects in ICH mouse model. The outcomes that we measured in this study have been utilized as standard outcome measurement in previously published ICH rodent model studies in which thrombin inhibition, microglia inactivation, and iron chelation were shown to have similar beneficial effects as observed in our study [26–28]. We evaluated the temporal expression of endogenous CTRP9 for 3 days after ICH. The results indicated that CTRP9 expression increased after ICH and reached the highest level at 24 h post-ICH. It has been reported that physiological levels of adiponectin in both human and mouse serum were elevated in response to inflammatory stimuli [29–32]. Our findings are consistent with a previous study that showed endogenous adiponectin expression significantly increased in cerebral ischemic areas after middle cerebral artery occlusion (MCAO) [33]. Acute injury or stress has been proposed to increase plasma concentration of elastase, which promotes the generation of globular adiponectin from full-length adiponectin [22, 34]. The acute stress following ICH injury may therefore contribute to increase endogenous CTRP9 expression after ICH. In addition, we observed that nasal administration of rCTRP9 increased the brain expression of CTRP9 and improved neurobehavioral deficits and brain edema after ICH. Even though the endogenous expression of CTRP9 was increased after ICH, it may not be sufficient to significantly reduce the massive neuroinflammatory insult after ICH. Exogenous administration of rCTRP9 further potentiates potential protective pathways against neuroinflammation after ICH.

Previous studies report that CTRP9 increased insulin sensitivity and lowered blood sugar levels in the hyperglycemia model [22]. Thus, we tested blood glucose changes during the first 24 h after rCTRP9 administration. We observed that intranasal administration of rCTRP9 did not affect blood glucose levels. Blood glucose in all the groups, including vehicle or rCTRP9 groups, reached highest levels at 1 h post-surgery, possibly a response to surgery-related stress. Additionally, subsequent blood glucose levels continued to decline in all groups, reaching the lowest levels at 24 h after ICH. These findings can possibly be attributed to surgery-induced stress and hematoma that increases energy expenditure as well as reduce food intake capacity of the animals. Since there were no difference observed in blood glucose levels between groups after intranasal rCTRP9 administration, it is reasonable to consider that rCTRP9 did not cause hypoglycemia in our ICH model.

We found that AdipoR1 was expressed not only in microglia but also in neurons and astrocytes by immunofluorescence staining. These results are consistent with previous reports. Although adiponectin receptors have been shown to exist abundantly in the brain, its exact role in brain diseases remains unclear. Currently, two adiponectin receptors have been identified, AdipoR1 and AdipoR2. The expression of AdipoR1 has been reported to be more pronounced than AdipoR2 in the brain [35]. In addition, AdipoR1 and AdipoR2 have differential binding affinities to various adiponectin multimers. AdipoR1 binds to globular adiponectin (gAd) with high affinity, while AdipoR2 has an intermediate affinity with both gAd and full-length adiponectin [36]. In addition, AdipoR1 predominantly binds to adiponectin and suppresses NFκB activation and subsequently reduces the expression of pro-inflammatory cytokine, while AdipoR2 does not mediate the effects of adiponectin on microglial cells [6]. In this study, we observed that intracerebroventricular injection of AdipR1 siRNA decreased the expression of AdipoR1 and worsened neurological function in naïve and ICH mice. These results indicated a beneficial role of AdipR1 as well as demonstrated the knockdown efficacy AdipoR1.

The expression of AdipoR1 is regulated by many factors; obesity, alcohol, and insulin resistance can lower while exercise, stress, and cerebral ischemic conditions can increase the levels of AdipoR1. Our findings showed that AdipoR1 expression temporarily increased after ICH and reached peak levels at 24 h post-ICH. The receptor AdipoR1 is a high-affinity receptor for globular adiponectin. An increase in AdipoR1 expression may be necessary to perform its function given the increase in expression of globular domain of CTRP9 after ICH injury. This may be a possible explanation for the increase in AdipoR1 expression after ICH. Additionally, our results showed that AdipoR1 was modulated in a similar manner to CTRP9 levels after ICH, which indicates that rCTRP9 may exert its function through AdipoR1. Furthermore, rCTRP9 administration increased the expression of AdipoR1. The results were consistent with previous study which showed that adiponectin increased the expression of AdipoR1 and protected the brain against cerebral ischemic stroke [37]. We speculate that acute ICH injury may initially activate the inflammasome pathway and then trigger the stress reaction of adiponectin AdipoR1 signaling to reduce local inflammation. This is supported by the findings of our study which demonstrated that CTRP9/AdipoR1 showed anti-inflammatory effects in the ICH model, which is consistent with a previous study in which adiponectin was protective against cerebral ischemic stroke [38]. We measured inflammatory cytokines expression in the brain after ICH and with rCTRP9 administration. Local immune cells such as microglia and peripheral immune cells have been implicated in contributing to neuroinflammation after ICH. Since we administered rCTRP9 via intranasal administration which allows direct delivery of the agent to the brain, we measured changes in cytokine expression in the brain and levels of circulating cytokines were not measured in this study. Furthermore, to demonstrate the role of AdipoR1 in the anti-inflammatory mechanism of rCTRP9, AdipoR1 siRNA was administered by intracerebroventricular injection at 48 h before ICH with rCTRP9 treatment. The results showed that knockdown of AdipoR1 abolished the protective effects of rCTRP9 and reversed the expression of downstream proteins, suggesting that AdipoR1 played an essential role in the activating the downstream anti-inflammatory pathway by rCTRP9.

Neuroinflammation is a key process leading to secondary neurological damage after ICH [39]. Although anti-inflammatory effects of adiponectin have been previously reported in many disease models [40–42], the underlying mechanism remains unclear. Next, we investigated the downstream signaling pathway mediated via AdipoR1 activation. APPL1 is the only adiponectin receptor (AdipoR)-interacting protein that has been identified. The NH2-terminal intracellular region of AdipoR directly interacts with the PTB domain of APPL1. APPL1 was required for AdipoR-induced activation of downstream pathways to exert its anti-inflammatory and cytoprotective effects [43]. Our results showed that APPL1 expression increased with rCTRP9 administration after ICH. AMPK is a major downstream signaling molecule activated by APPL1 and has been demonstrated by studies on adiponectin-dependent AMPK signaling. A recent study suggested that bakkenolide B inhibited lipopolysaccharide-induced pro-inflammatory cytokines via AMPK activation in microglia [44]. In our study, administration of rCTRP9 markedly increased the expression of p-AMPK. This finding was consistent with a previous study in which CTRP9 protected against acute cardiac injury following ischemia-reperfusion via AMPK-dependent mechanism [45]. Due to its role in inducing the expression of proinflammatory cytokines, the nuclear factor NFκB pathway has long been considered as a typical proinflammatory signaling pathway. Prior published studies showed that the anti-inflammatory effects of adiponectin may be mediated via AMPK/NFκB signaling pathway [46]. In this study, we observed that rCTRP9 treatment upregulated the expression of AdipoR1, APPL1, and p-AMPK, and downregulated the pro-inflammatory p-NFκB after ICH. Furthermore, AdipoR1 siRNA and Dorsomorphin reversed the anti-inflammatory effects by rCTRP9. Overall, our results indicated that rCTRP9 treatment exerted anti-inflammatory effects through AdipoR1/AMPK/NFκB pathway in ICH model.

There are some limitations in the present study. First, AdipoR1 is expressed in astrocytes and may play a role in blood-brain barrier protective effects of rCTRP9, which may also contribute to the beneficial effects of rCTRP9 after ICH. Second, the pathophysiology of neuroinflammation after ICH is a complex network. Other downstream factors such as CAMKKβ and PPARα may be modulated by rCTRP9 administration after ICH which was not explored in this study. In the current study, we focused on APPL1/AMPK signaling anti-inflammatory mechanisms. Further studies need to be conducted to explore other potential signaling pathways contributed by rCTRP9 and AdipoR1 activation. Third, we did not evaluate the effects of CTRP9 in different age groups or in ICH with systemic co-morbidities. ICH tends to occur in older population with hypertension, vascular disorders, cerebral amyloid angiopathy, or anticoagulation. Previous publications indicate that adiponectin may have effect on hypertension and aging. Clinical studies have shown a relationship between serum adiponectin concentrations and the activity of renin-angiotensin-aldosterone system (RAAS), leading to changes in blood pressure [47, 48]. The effect of adiponectin on the cardiovascular system has been demonstrated partly by activation of the AMPK and cyclooxygenase-2 (COX-2) pathways, relaxation of vascular endothelial cells, reduction of endothelial cell apoptosis, and promotion of nitric oxide production [49, 50]. The lifespan of AdipoR1 or AdipoR2 knockout mice was reported to be shorter than that of control mice, the mechanisms involved included AMPK, mammalian target of rapamycin (mTOR) and SIRT, which are known to be key longevity molecules [51]. Overall, these findings indicate a need to study the relationship between adiponectin and CTRP9 with hypertension, aging, and systemic co-morbidities associated with ICH. Fourth, in this study, we did not evaluate sex-specific differences in the effects of CTRP9 in ICH. Estrogen has been reported to have neuroprotective effects, and it reduced neurological impairment after ICH and subarachnoid hemorrhage in rats [52, 53]. We therefore, used 8-week-old male adult CD1 mice for this study in order to eliminate the interference due to the effects of female sex hormones in the study outcomes.

## Conclusions

In summary, we first showed that rCTRP9 administration improved neurological function, reduced brain edema, and alleviated inflammation through AdipoR1/AMPK/NFκB pathway after ICH. CTRP9, the closest analogue of adiponectin, may be a potential therapeutic target against neuroinflammation after ICH.

## Additional files

Additional file 1: **Figure S1.** Experimental design and animal groups. ICH, intracerebral hemorrhage; rCTRP9, recombinant C1q/TNF-related protein 9; WB, western blot; IHC, immunohistochemistry; BS, blood sugar; siRNA, small interfering ribonucleic acid. (TIF 2484 kb)

Additional file 2: **Table S1.** Summary of experimental groups and mortality rate in the study. (DOCX 21 kb)

Additional file 3: **Figure S2.** Schematic mechanism of the effects of rCTRP9 on anti-neuroinflammation after ICH. rCTRP9, recombinant C1q/TNF-related protein 9; AdipoR1, adiponectin receptor 1; APPL1, adaptor protein, phosphotyrosine interacting with PH domain and leucine zipper 1; p-AMPK, phosphorylated-adenosine monophosphate-activated protein kinase; p-NFκB, phosphorylated-nuclear factor kappa B; TNFα, tumor necrosis factor α; IL-6, interleukin-6. (TIF 387 kb)

## Abbreviations

AdipoR1: Adiponectin receptor 1
AMPK: Adenosine monophosphate-activated protein kinase
APPL1: Adaptor protein, phosphotyrosine interacting with PH domain and leucine zipper 1
BWC: Brain water content
CTRP9: C1q/TNF-related protein 9
DMSO: Dimethyl sulfoxide
GFAP: Glial fibrillary acidic protein
Iba-1: Ionized calcium binding adaptor molecule-1
ICH: intracerebral hemorrhage
IL-6: Interleukin-6
NeuN: Neuronal nuclear
p-AMPK: Phosphorylated-AMPK
PBS: Phosphate-buffered saline
p-NFκB: Phosphorylated-nuclear factor kappa B
Scr siRNA: Scramble siRNA
siRNA: Small interfering ribonucleic acid
TNFα: Tumor necrosis factor
