# Supplementary figures and images for: Recombinant CTRP9 administration attenuates neuroinflammation via activating adiponectin receptor 1 after intracerebral hemorrhage in mice
Source: J Neuroinflammation. 2018 Jul 30;15:215. doi: 10.1186/s12974-018-1256-8 (PMC6066941; doi:10.1186/s12974-018-1256-8)

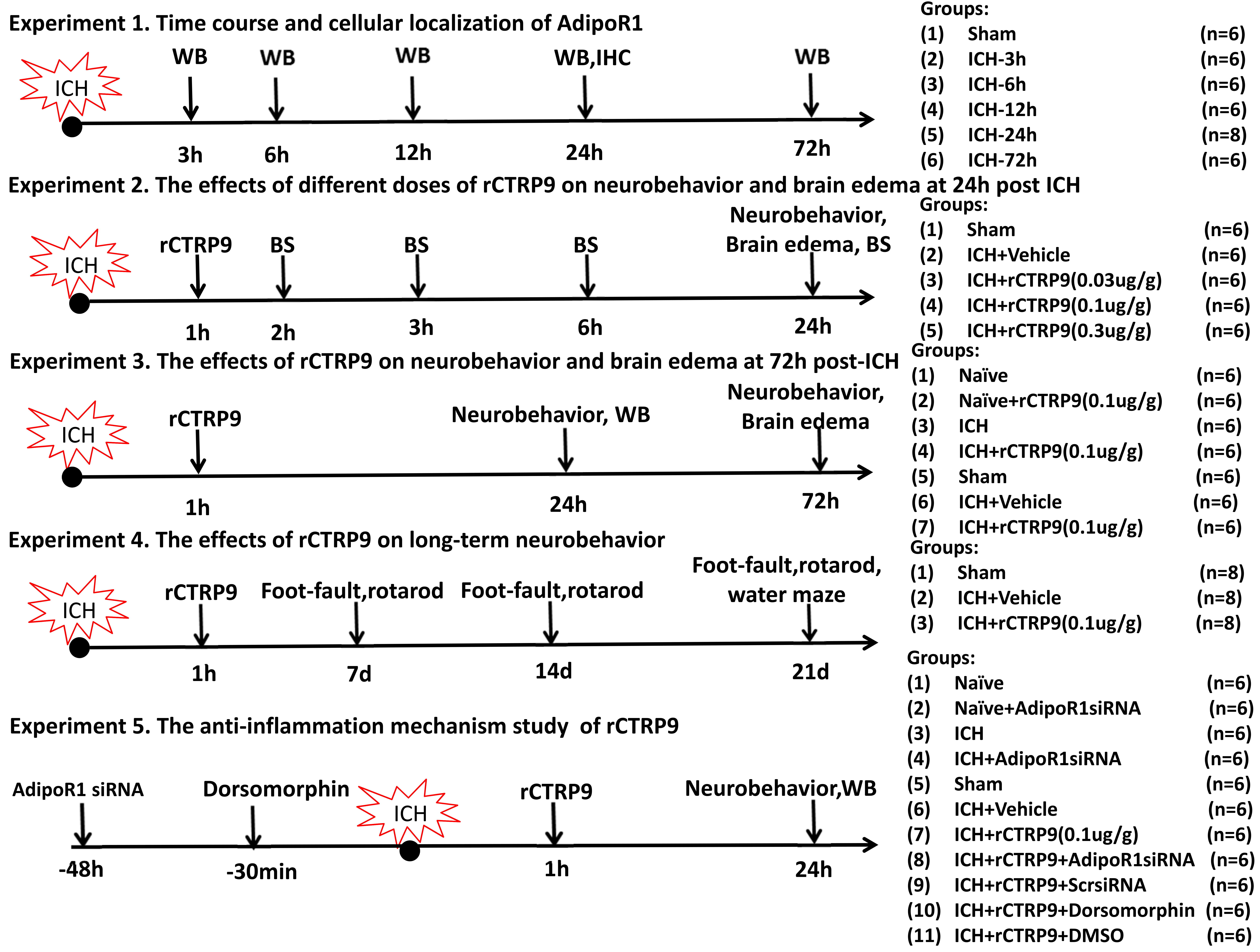

Supplement: Supplementary file 1 — Figure S1. Experimental design and animal groups. ICH, intracerebral hemorrhage; rCTRP9, recombinant C1q/TNF-related protein 9; WB, western blot; IHC, immunohistochemistry; BS, blood sugar; siRNA, small interfering ribonucleic acid. (TIF 2484 kb) [file 12974_2018_1256_MOESM1_ESM.tif]

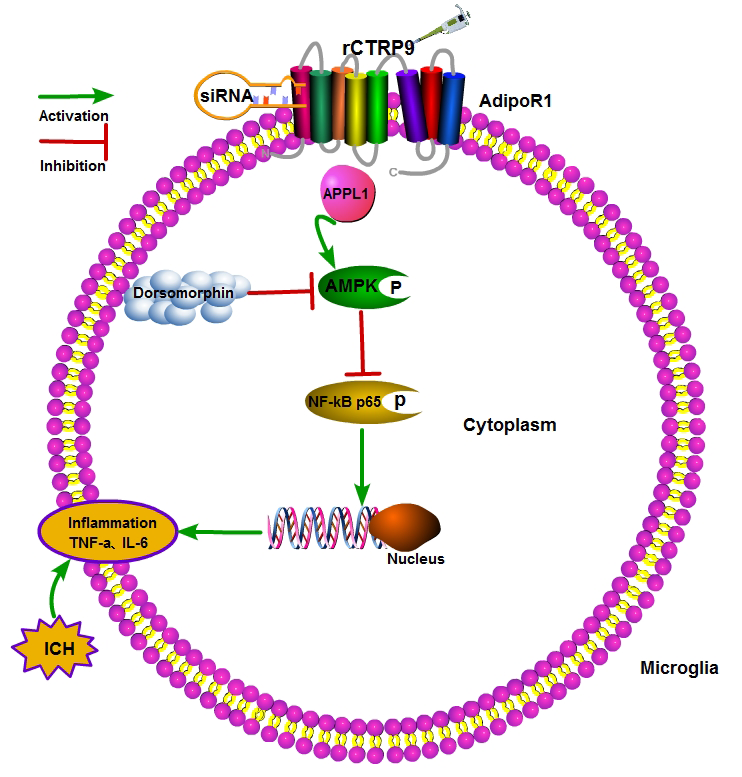

Supplement: Supplementary file 3 — Figure S2. Schematic mechanism of the effects of rCTRP9 on anti-neuroinflammation after ICH. rCTRP9, recombinant C1q/TNF-related protein 9; AdipoR1, adiponectin receptor 1; APPL1, adaptor protein, phosphotyrosine interacting with PH domain and leucine zipper 1; p-AMPK, phosphorylated-adenosine monophosphate-activated protein kinase; p-NFκB, phosphorylated-nuclear factor kappa B; TNFα, tumor necrosis factor α; IL-6, interleukin-6. (TIF 387 kb) [file 12974_2018_1256_MOESM3_ESM.tif]
