# Supplementary material for: Recombinant CTRP9 administration attenuates neuroinflammation via activating adiponectin receptor 1 after intracerebral hemorrhage in mice
Source: J Neuroinflammation. 2018 Jul 30;15:215. doi: 10.1186/s12974-018-1256-8 (PMC6066941; doi:10.1186/s12974-018-1256-8)
Supplement: Supplementary file 2 — Table S1. Summary of experimental groups and mortality rate in the study. (DOCX 21 kb) [file 12974_2018_1256_MOESM2_ESM.docx]

| **Experimental Groups** | **Neurobehavioral test** | **IHC** | **WB** | **Exclusion** | **Mortality** | **Subtotal** |
| --- | --- | --- | --- | --- | --- | --- |
|  | **BWC** |  |  |  | **(%)** |  |
| **Experimental 1** |  |  |  |  |  |  |
| Sham |  | 0 | 6 | 0 | 0 | 6 |
| ICH (3h, 6h, 12h, 24h, 72h) |  | 2 | 30 | 3 | 2(5.7%) | 35 |
| **Experimental 2** |  |  |  |  |  |  |
| Sham | 6 |  |  | 0 | 0 | 6 |
| ICH + Vehicle | 6 |  |  | 1 | 1(14.29%) | 7 |
| ICH + rCTRP9 (0.03μg/g) | 6 |  |  | 1 | 1(14.29%) | 7 |
| ICH + rCTRP9 (0.1μg/g) | 6 |  |  | 1 | 0 | 7 |
| ICH + rCTRP9 (0.3μg/g) | 6 |  |  | 0 | 0 | 6 |
| **Experimental 3** |  |  |  |  |  |  |
| Naive |  |  | 6 | 0 | 0 | 6 |
| Naive + rCTRP9 |  |  | 6 | 0 | 0 | 6 |
| ICH |  |  | 6 | 1 | 1(14.29%) | 7 |
| ICH + rCTRP9 |  |  | 6 | 0 | 0 | 6 |
| Sham | 6 |  |  | 0 | 0 | 6 |
| ICH + Vehicle | 6 |  |  | 1 | 1(14.29%) | 7 |
| ICH + rCTRP9 (0.1μg/g) | 6 |  |  | 0 | 0 | 6 |
| **Experimental 4** |  |  |  |  |  |  |
| Sham | 8 |  |  | 0 | 0 | 8 |
| ICH + Vehicle | 8 |  |  | 2 | 2(20%) | 10 |
| ICH + rCTRP9 (0.1μg/g) | 8 |  |  | 1 | 1(11.1%) | 9 |
| **Experimental 5** |  |  |  |  |  |  |
| Naive |  |  | 6 | 0 | 0 | 6 |
| Naïve+AdipoR1 siRNA |  |  | 6 | 0 | 0 | 6 |
| ICH |  |  | 6 | 1 | 1(14.29%) | 7 |
| ICH+AdipoR1 siRNA |  |  | 6 | 2 | 2(25%) | 8 |
| Sham |  |  | 6 | 0 | 0 | 6 |
| ICH + Vehicle |  |  | 6 | 2 | 1(12.5%) | 8 |
| ICH + rCTRP9 (0.1μg/g) |  |  | 6 | 1 | 1(14.29%) | 7 |
| ICH + rCTRP9 + AdipoR1 siRNA |  |  | 6 | 0 | 0 | 6 |
| ICH + rCTRP9 + Scr siRNA |  |  | 6 | 0 | 0 | 6 |
| ICH + rCTRP9 + Dorsomorphin |  |  | 6 | 1 | 1(14.29%) | 7 |
| ICH + rCTRP9 + DMSO |  |  | 6 | 0 | 0 | 6 |
| **Total** | 72 | 2 | 126 | 18 | 15(9.2%) | 218 |

Table S1: Summary of experimental groups and mortality rate in the study.

ICH, intracerebral hemorrhage; BWC, brain water content; WB, western blot; IHC, immunohistochemistry; siRNA, small interfering ribonucleic acid; DMSO, dimethyl sulfoxide

A total of 218 mice were used of which 56 were sham and 162 mice underwent ICH induction. None of the sham mice died and the mortality rate in ICH group was 9.2% (15/162).
